# Cambodia's Care Services for Individuals With Disabilities (Hearing Loss and Ear Disease): A Perspective on Challenges and the Urgent Need for Improvement

**DOI:** 10.1002/hsr2.72200

**Published:** 2026-03-26

**Authors:** Virak Sorn, Sokhoeun Eat

**Affiliations:** ^1^ Faculty of Health Sciences and Biotechnology University of Puthisastra Phnom Penh Cambodia

**Keywords:** Cambodia, challenges, disabilities, ear and hearing loss care, health system

## Abstract

**Background and Aim:**

In Cambodia, little is known or paid attention to regarding the care services among disabled people, especially conditions of ear disease and hearing loss care, or the roles that key stakeholders or providers play in providing services. This perspective article aims to discuss and highlight the challenges and critical needs for improvement to address the issues more effectively.

**Methods:**

A search on Google Scholar was carried out to identify relevant journal articles. Additionally, relevant reports and data on the care of ear disease and hearing loss were sourced from the websites of international and humanitarian organizations, including the World Health Organization, the Ministry of Health of Cambodia, and other relevant sources.

**Results:**

Cambodia's disability care services have improved but still face challenges in implementation, funding, and resource allocation. The country should focus on four key priorities for ear and hearing care: integrating services into primary care, developing infrastructure, ensuring sustainable financing, and implementing effective services at all levels. Addressing stigma and public awareness, strengthening multisectoral collaboration, increasing financial investment, and promoting inclusive policies can help improve services and universal health coverage for people with disabilities.

**Conclusions:**

Care of individuals with disabilities (ear disease) presents a major public health challenge for Cambodia, which is made worse by delays, a lack of coordination, and a shortage of infrastructure and personnel. To solve this, it is necessary to incorporate ear and hearing care into primary healthcare services, upgrade infrastructure, provide professional training, and set up long‐term funding sources.

## 1 Introduction

Disabled people, especially those with ear disease and hearing loss, experience broad and profound effects on a person's well‐being, education, socioeconomic opportunity, and quality of life [1]. Hearing loss is the most common sensory impairment, which has impacted over 1.57 billion people worldwide who are thought to have mild or worse hearing loss [2]. Individuals with hearing disabilities are associated with premature cognitive decline, depression, an increased risk of dementia, poorer balance, falls, hospitalizations, and early mortality [3]. The distribution of ear disease and hearing loss is highly unequal, and locations have limited access to ear and hearing care, with people living in low‐ and middle‐income countries (LMICs) bearing 80% of the global burden [2, 3, 4, 5].

In Cambodia, the public, private, and nongovernmental sectors all offer ear and hearing care; however, access is restricted by a severe lack of providers, facility locations, equipment, and resource shortages, and a lack of policy implementation for hearing‐related disabilities and rehabilitation [2]. According to the previous report, there are nine public hospitals with ear, nose, and throat (ENT) departments across the country, with five located in the capital city. Private hospitals have two ENT departments, and nongovernmental organizations with international connections provide speciality surgery, aural rehabilitation, and diagnostic audiology for chronic ear conditions [1]. To achieve universal health coverage (UHC), primary healthcare might be a useful instrument that lessens the strain on more advanced medical institutions [6]. A key component of delivering cost‐effective interventions into mainstream health services is strengthening the health system by incorporating ear and hearing care into the primary healthcare framework and using task‐sharing approaches for community health workers in the identification, prevention, and rehabilitation of hearing loss [7, 8, 9]. Though it involves many organizations, stakeholders, providers, and interventions, the process of integrating care into primary healthcare is complicated, and specific integration techniques are still unclear [9, 10]. According to the World Health Organization's (WHO) vision, clinical ear and hearing care services are to be accessible and integrated within national health services and delivered across all levels of care, which is the lofty objective established and achieved by every nation [11, 12].

Currently, little is known about ear and hearing care services in Cambodia, and the potential contributions of key stakeholders or providers to the integration of ear and hearing care services into primary healthcare are not well understood. Therefore, this perspective article aims to discuss and highlight the challenges and critical needs for improvement to address the issues more effectively. By advancing knowledge of ear and hearing care practices, this information may help shape public policies and initiatives that promote the delivery of services that address health inequalities and enhance the health of individuals with disabilities, especially those with ear or hearing disorders.

## 2 Current Challenges

In Cambodia, care services for disabled people have improved over the past three decades, and efforts are ongoing to improve the quality of life for those with disabilities. However, along with the improvement, there are challenges, including the barriers in access to healthcare services, the duration and severity of disease, and the lack of adequate services, which pose a significant public health challenge among people with disabilities [7], especially those who have ear and hearing loss [4, 5]. Since there are disability services provided at the rural healthcare for health services, the disabled people are faced with concerns about the received quality of care and access to the care services [10]. According to Waterworth et al. [2], the survey has illustrated that adults accounted for 78% of the total 108 participants (51% male and 49% female), while 22% were caregivers of children (64% male and 36% female). The results showed that the most common difficulty experienced was the lack of local ear‐care services, reported by 75% (81/108) of participants; 57% (62/108) cited lack of knowledge about service availability as a barrier to obtaining prior care, and 12% (13/108) reported having no knowledge of local ear‐care services. Participants living in rural or remote areas also faced travel‐related barriers, with journeys lasting up to 8 h, regardless of the mode of transport available to them [2]. As illustrated by Kleinitz et al. [13], the barriers to access to health services for people with disabilities are influenced by several factors, including finances, quality of care, people with disabilities having poor knowledge, sociocultural negative beliefs and attitudes associated with disability, and long distances to health facilities and long waiting times to get treatment [13]. There are multiple factors that contributed to the delays in seeking care at a specialist center, in addition to the historic lack of service availability across all levels of the healthcare system in the country. These factors included a perceived lack of knowledge about hearing loss and ear disease, including its causes and/or treatments, as well as a lack of awareness about treatment from an expert [14, 15]. On the other hand, most patients reported a lack of ear and hearing care services at the primary care level and even fewer specialist services at the secondary or tertiary level, and they resorted instead to simple medical care [16].

In Cambodia, the Ministry of Health (MoH) oversees all mainstream health services; however, the Ministry of Social Affairs, Veterans, and Youth Rehabilitation (MoSVY) is in charge of matters pertaining to disabilities. Since the two ministries do not currently coordinate well, this affects the standard and continuity of care given to individuals with disabilities, especially for those living in both rural and urban areas [15]. The lack of awareness of the causes of hearing problems and the lack of knowledge of ear and hearing care services likely reflect the fact that such services are simply not available locally, as evidenced also in feelings of fear, stigma, and a lack of trust in providers [2]. Patients sought care where it was locally available, as shown by their prior treatments. The report also illustrated that even in places lacking professional services or high‐quality healthcare, they were prepared to pay hefty out‐of‐pocket expenses (between $500 and $600 USD), which was a substantial sum in comparison to average incomes, in order to receive care [1, 2]. Similarly, Grundy and Annear [16], most of the patients prefer to visit private clinics/providers, which reflects a general belief that they provide a better quality of care than public services.

Overall, healthcare professionals lack understanding of the causes and management of otitis media, as well as the inability to coordinate care among providers within the healthcare system. They show a significant burden of ear disease in Cambodia, which is related to delays in receiving timely and effective treatment and exceeds the current workforce and infrastructural capacity in both the public and private sectors. As healthcare facilities were generally unavailable in rural areas, people tended to “get by,” learning to live with their symptoms for a significant period of time. Patients continued to experience “ear health problems” and became heavily conditioned as a result of the low quality of care provided by the health facilities that specialize in ear and hearing care, which in turn exacerbated their chronic symptoms [2, 16].

## 3 Perspectives on Care Services for Individuals With Disabilities

Although Cambodian care services for people with disabilities have steadily improved, there are still significant gaps in terms of inclusion, quality, and accessibility. Through initiatives like the National Disability Strategic Plan and the creation of the Disability Action Council, the Cambodian government has worked to assist people with disabilities [17]. Despite efforts, challenges remain in terms of implementation, funding, and resource allocation for disability care services within the existing healthcare system. Receiving appropriate treatment for people with disabilities, especially those with hearing loss and ear diseases are made more difficult by the fact that several medical facilities still lack specialized services, professional staff, and necessary equipment to assist patients [11, 12]. Therefore, as policymakers shape future health strategies, Cambodia should focus on four key priorities for ear and hearing care. Those strategies include (a) integrating services into primary care; (b) developing infrastructure and training healthcare professionals; (c) ensuring sustainable financing and social protection mechanisms; and (d) ensuring effective implementation of the services at all levels of care [1, 2, 18].

Through appropriate referral pathways for patients with disabilities, policies that guarantee the integration of high‐quality ear and hearing care into primary care services can address disease prevention, diagnosis, treatment, and management, as well as overall health promotion. The global evidence suggests that integrating ear and hearing care within primary care through patient education campaigns, improved health insurance coverage, investment in provider training, and the promotion of digital health technologies can significantly improve access in rural or remote areas [19, 20]. This is consistent with the WHO's report, which recommended prioritizing a strategy of integrated community‐based and people‐centered for ear and hearing care to improve local access to interventions and to improve coordination of referral pathways [11]. Regarding ear and hearing care infrastructure, improving hearing care in government health facilities can be achieved by strengthening both the minimum and complementary packages of activities provided at government facilities [21, 22]. This would also serve the purpose of providing patient benefit through existing social health protection mechanisms such as the health equity funds and newly proposed social insurance arrangements [2]. Recent health system strengthening in Cambodia has created a foundation for better addressing the growing burden of noncommunicable diseases [23], including the need for improved ear and hearing care.

The daily lives of people with disabilities are still impacted by stigma and a lack of public awareness, according to societal perspectives. Many continue to experience prejudice in workplaces, schools, and healthcare environments. Access to healthcare services that are disability‐friendly is even more limited in rural locations, leading to delays in diagnosis and treatment. Long‐term sustainability is still an issue, even if NGOs and international organizations are essential in addressing service gaps. To guarantee that people with disabilities receive the care and assistance they require to live independent and respectable lives, it is imperative to strengthen multisectoral collaboration, increase financial investment, and promote inclusive policies. By addressing these challenges, there will not only be better services provided to the people with disabilities; however, it will also impact the achieved UHC in the country [24]. This presents an opportunity to more effectively tackle the personal, healthcare, and economic impacts on patients with hearing loss.

## 4 Conclusion

In conclusion, providing care for people with disabilities (ear diseases) poses a significant public health challenge in Cambodia, with the situation worsened by delays in accessing timely and effective treatment. This issue is further compounded by a lack of coordination among healthcare providers and insufficient workforce and infrastructure capacity. While Cambodia's MoH strives to improve the availability, quality, and affordability of government health services, integrating ear and hearing care services into the health system remains a persistent challenge. To address this, policymakers should focus on three key areas: integrating ear and hearing care into primary healthcare services, improving infrastructure and training healthcare professionals, and establishing sustainable financing and social protection mechanisms for people with disabilities. Tackling these challenges requires a comprehensive, coordinated effort involving the government, private sector, NGOs, and the active participation of citizens.

## 5 Recommendation

To further strengthen the public health system and provide better services to people with disabilities, the country needs to ensure continuous professional development for healthcare providers at all levels nationwide. Adopting policies that integrate quality care for people with disabilities into primary care can create a continuum of care, encompassing health promotion, diagnosis, treatment, and disease management. Strengthening the minimum and complementary packages of activities offered at government facilities can also benefit patients through existing social health protection mechanisms among those with disabilities. Encouraging community involvement and improving communication systems can help UHC reach its goals and improve even more.

## Author Contributions

**Virak Sorn:** conceptualization, data curation, formal analysis, writing – original draft, writing – review and editing. **Sokhoeun Eat:** data curation, formal analysis, writing – original draft, writing – review and editing. All authors have read and approved the final version of the manuscript. Virak Sorn (corresponding author) had full access to all of the data in this study and takes complete responsibility for the integrity of the data and the accuracy of the data analysis.

## Conflicts of Interest

The authors declare no conflicts of interest.

## Transparency Statement

The lead author Virak Sorn affirms that this manuscript is an honest, accurate, and transparent account of the study being reported; that no important aspects of the study have been omitted; and that any discrepancies from the study as planned (and, if relevant, registered) have been explained.

## Data Availability

Due to the fact that no data sets were created or examined for this article, data sharing is not applicable.
